# Endometriosis: advances and controversies in classification, pathogenesis, diagnosis, and treatment

**DOI:** 10.12688/f1000research.14817.1

**Published:** 2019-04-23

**Authors:** Edgardo Rolla

**Affiliations:** 1 Sociedad Argentina de Endometriosis, Buenos Aires, Argentina; 2 Sociedad Argentina de Cirugía Laparoscópica, Buenos Aires, Argentina; 3 World Endometriosis Society, Buenos Aires, Argentina

**Keywords:** endometrosis deep infiltrating endometriosis pain infertility

## Abstract

Endometriosis is an enigmatic disease that could start at birth. Its pathogenesis is supported by different theories. Accumulating facts relate it to a multigenic disorder. In this review of recent publications, the principal symptoms of the disease, pain and infertility, as well as its pathogenesis, diagnosis, and classification will be addressed. Endometriosis presents three main variants: superficial peritoneal disease, deep infiltrating endometriosis, and ovarian endometriomas. The management of the disease, surgery, and medical and alternative therapies will be discussed. Special reference will be made to the quality of surgery and how to understand patients with endometriosis and endometriosis.

## Introduction

This is not a meta-analysis. It is an opinion article based on an extensive search of recent literature, an update of known facts enhanced by novel investigations.

Endometriosis is an enigmatic disease of yet-unknown origin and pathogenesis. It is sustained by theories from long ago, when Sampson ^1^ described it as ectopic implants of menstrual shredding passed to the abdominal cavity through the Fallopian tubes. Recently, Brosens and Benagiano ^2^ suggested that it starts with neonatal hormonal deprivation bleeding that many newborn girls express in a retrograde fashion. Implants would remain until puberty.

A celomic theory states that embryonic cells from the Müllerian ducts persist in ectopic locations. At puberty, stimulated by estrogens, they grow to build up endometriotic lesions ^3^.

According to Nyholt *et al*. ^4^, endometriosis is a “heritable, hormone-dependent gynecological disorder”. In their meta-analysis, they identified five novel loci related to the risk of developing endometriosis. All five are involved in sex steroid pathways.

There is no reliable serum maker for this disease, and imaging still leaves much of it undiagnosed. Ultrasound (US) has a good sensitivity and specificity for endometriomas (83% and 89%, respectively) ^5^. Unfortunately, in the case of deep infiltrating endometriosis (DIE), uterosacral ligaments, rectovaginal septum, vagina, and bladder, the overall pooled sensitivity and specificity of US transvaginal studies (TVSs) range between 53% and 93% ^6^.

High-resolution magnetic resonance imaging (MRI) with bladder, vaginal, and rectal contrast has been a breakthrough in recent times, as we proved at the recent XIII World Congress on Endometriosis ^7, 8^.

Is there a psychological trait common to women with endometriosis? Several publications search for predictors of psychological distress ^9^ but few focus on the personality of patients with endometriosis. Even fewer identify associations with their psychological aspects. On patient health questionnaires, women with endometriosis show a high frequency of positive results for psychiatric disorders significantly associated with pain severity ^10^. No personal traits allow us to identify subjects prone to develop endometriosis.

Laparoscopy is the “gold standard” for the diagnosis of endometriosis. Surgical biopsies allow histological confirmation. Laparoscopy should be performed preferably by experienced surgeons. Removal of all disease present must be accomplished in the same procedure. The recent World Endometriosis Society (WES) Consensus for the Current Management of Endometriosis ^11^ states that “Individualized care benefits from a multi-disciplinary network of experts sufficiently skilled in providing advice on, and treatment of endometriosis and its associated symptoms, based on the best available knowledge, their extensive experience and their transparent record of success rates”.

An ongoing discussion, the classification of endometriosis, will be thoroughly reviewed in this article.

Medical management is a centerpiece. According to the Consensus, old-time favorites such as danazol or gestrinone should be used only in the absence of side effects when other treatments have proven ineffective ^11^. Progestagens have proven efficacy ^12^, whereas gonadotropin-releasing hormone (GnRh) agonist therapy is not recommended for long-term use. Oral progestin-only pills have demonstrated their ability to control the extent of endometriotic lesions on a long-term basis ^13^.

Combined oral contraceptives (OCs) provide initial pain relief, but the long-term efficacy as a treatment for endometriosis lacks clinical evidence ^13^. Moreover, there are even some data supporting potential adverse effects on the progression of the disease.

Ulipristal entails rare but severe risks such as endometrial hyperplasia ^14^, endometrial carcinoma ^15^, and hepatic damage. As recently as August 2018, the US Food and Drug Administration (FDA) had not approved the use of this drug for the treatment of myomas (and endometriosis as well). In June 2018, the European Medical Agency (EMA) approved its use as a preoperative treatment of fibroids. This short-term use before surgery could be considered for endometriosis.

Newly introduced oral GnRh antagonist elagolix NR is associated with few minor side effects (hot flashes), excellent reduction of endometriosis-associated pain, and arrest of the progression of the disease when used for an extended period of 12 months ^16, 17^ at a 200-mg daily dose. Some moderate detrimental effects on bone density were reported ^18^, suggesting that this drug should be used with a hormonal backup.

Surgery should be considered, during laparoscopy, in the treatment of the disease. All lesions present should preferably be resected. The issue of endometriomas, a never-ending dilemma, is discussed with sound evidence from recent literature.

Infertility treatments for patients with endometriosis need special consideration. Surgery and assisted reproduction techniques (ARTs) cross over according to the different stages of the disease and the patient’s age. Minimal and mild disease frequently benefit from expert surgery. Advanced moderate and severe stages usually require *in vitro* fertilization (IVF).

DIE should be treated only by expert surgeons, preferably by interdisciplinary teams. The question of whether it should be operated before infertility treatments remains controversial.

Quality of surgery and certification of expertise are being revised by the Consensus group of the WES. This issue will be thoroughly reviewed in the present publication.

Understanding endometriosis (and patients with endometriosis) is the closing item. Current opinions on endometriosis quality of life (QOL) and psychosocial effects of the disease will be discussed. Do not attempt to find in this publication a meta-analysis or systematic review of the literature. On the contrary, a documented update of endometriosis will be presented.

## Pathogenesis

According to a recent review ^19^, there is growing evidence that hormonal and immune factors create a pro-inflammatory microenvironment that facilitates the persistence of endometriosis. This relates to the disease’s two main symptoms: pain and infertility. New drugs on the market (and in research) have pharmacological effects on the endocrine and inflammatory functions implicated in the pathogenesis of the disease. This will lead to new investigative pathways in the pathogenesis of endometriosis.

### A. Implantation theory

In 1927, Sampson ^1^ proposed a retrograde flow of the menstrual mix of blood and full endometrial tissue through the Fallopian tubes into the peritoneal cavity as the first step in the development of the disease. Brosens and Benagiano ^2^ suggest that the first retrograde bleeding occurs at birth, when the newborn girl has drastic hormonal deprivation. Tight internal uterine cervix os, thick cervical mucus, or malformations impede the normal external drainage of that mixture, which Brosens and Benagiano consider a source of stem cells. This results in the passage of that content into the abdominal cavity. These first implants will remain dormant because of the lack of estrogens in childhood. They shall grow rapidly after puberty, when the ovaries start to produce sexual hormones.

### B. Celomic theory

According to Burney and Giudice ^3^, “celomic metaplasia involves the transformation of normal peritoneal tissue to ectopic endometrial tissue”. Endocrine-disrupting chemicals might play an important role in such transformation. Addressing the theory of Müllerian rests, the authors state that residual cells from the embryonic Müllerian duct migration “maintain the capacity to develop into endometriotic lesions under the influence of estrogens” ^3^.

Endocrine, immune, and stem/progenitor cells and epigenetic modifications “must be considered in the context of genetic background as well as stimulus driven reprogramming of the female reproductive tract” ^3^. Even extrauterine stem/progenitor cells derived from bone marrow are suggested to be possible sources of ectopic endometriotic tissue ^20^.

### C. Inflammatory disease

Dmowski (cited by Burney and Giudice ^3^) suggests that there is evidence that endometriosis is, in fact, a pelvic inflammatory condition. A “peritonitis without germs”? The peritoneal fluid has an increased concentration of activated macrophages and an inflammatory profile in the cytokine/chemokine axis. Zimmer, in the review by Burney and Giudice, is reported to link a haptoglobin-like protein (that binds macrophages and reduces their phagocytic capacity) to the genesis of endometriosis. Increased production of interleukin-6 (IL-6), macrophage migration inhibitory factor, tumor necrosis factor-alpha, IL-1b, IL-6, and IL-8 alterations is also described. Gargett *et al*. ^21^ propose that human endometrium regenerates cyclically every month mediated by endometrial stem/progenitor cells such as CD140b ^+^, CD146 ^+^, or SUSD2 ^+^ endometrial mesenchymal stem cells (eMSCs). N-cadherin ^+^ endometrial epithelial progenitor cells and side population cells would also contribute to the pathogenesis of the disease. They are planted retrogradely at the time of birth or at puberty. The authors propose that the eMSCs may have a role in the generation of progesterone-resistant phenotype endometrial stromal fibroblasts. Stem/progenitor cell differences between healthy women and those with endometriosis have been proven.

### D. Endometriomas

On the genesis of endometriomas, several theories have been updated by Rizzello and Coccia ^22^:

1. Invagination: They are only pseudocysts produced by the accumulation of menstrual debris, which include active implants at the site of inversion.
2. Celomic metaplasia: They originate from invaginated ovarian celomic epithelium, which has metaplasia of its glandular epithelium and stroma.
3. Follicular: Some researchers proposed that endometriomas could originate from ovarian follicles, but no clear explanation for this theory was ever given.

### E. Aromatases

A rare case of postmenopausal hepatic flexure colon DIE, in a woman who had previously undergone total hysterectomy and bilateral salpingooforectomy, was recently presented by Snyder *et al*. ^23^. They propose that autologous aromatase production at the site gave rise to a full-thickness infiltrating nodule from remnants or metaplastic endometrial tissues. They considered this after checking that, without any surgery at all, the nodule vanished after prolonged anti-aromatase treatment.

### F. Hormonal receptors

Nisolle *et al*. ^24^ found “heterogeneity of estrogen receptor α and progesterone receptor distribution in lesions of DIE in untreated women, or during exposure to various hormonal treatments”. This could be because DIE nodules are poor responders to different endocrine treatments.

### G. Deep infiltrating endometriosis

Petraglia and Chapron ^25^ consider DIE a different phenotype of the same disease, shared with endometriomas and peritoneal lesions. It includes two locations: anterior compartment disease (bladder) and posterior compartment disease (vagina, uterosacral ligaments, rectum, and ureters).

Some invasive mechanisms characteristic of endometriosis, such as the expression of matrix metalloproteinases and activins, are enhanced in DIE. Also, a very high expression of the different mechanisms of neuroangiogenesis (nerve growth factors, vascular endothelial growth factor, and intercellular adhesion molecule) is present.

For them, other immunological factors (peritoneal macrophages, natural killer cells, and lymphocytes) are critically altered in DIE. The aggressive behavior of DIE may be explained by the highly decreased apoptosis. Nuclear factor kappa-light-chain-enhancer of activated B cells (NF-κB), B-cell lymphoma 2 (Blc-2), anti-Müllerian hormone, and the increased proliferation activity related to oxidative stress (NF-κB, reactive oxygen species, extracellular regulated kinase, and advanced oxidation proteins) also contribute.

### H. Epigenetic modulators

In a recent “master review”, Gordts, Koninckx, and Brosens elaborate two different pathogenic hypotheses ^26^:

Hypothesis I: pathogenesis of early-onset endometriosis by neonatal uterine bleeding with the cyclic menstruation is the driving mechanism for adenomyotic nodule formation.

Hypothesis II: deep endometriosis is a specific type of abnormal endometrium-like cell benign tumor.

As proof of this, the authors show that deep endometriosis is a specific disease, as reflected by the distribution of deep lesions in all stages of the Revised American Fertility Society classification.

It might share with peritoneal or cystic endometriosis the same cellular origin, but genetic and epigenetic modulators induce distinct presentations of the disease. In some cases, peritoneal endometriosis will prevail. With other epigenetic modulators, DIE will grow.

They have common structures when analyzed by the pathologist. These authors propose that uterine adenomyosis and DIE have common origins, as in both cases glands are seen infiltrating muscle tissue.

## Diagnosis

### A. Anamnesis

Listen to the patient. Carry on a detailed anamnesis in a very slow fashion. This simple action gives us the best approach to the disease. She has so much to tell, to show with her face and expression. In most cases, the disease can be understood just by listening.

The omnipresent symptom is pain: cyclic pelvic pain, dysmenorrhea, periovulatory pain, chronic non-cyclic pelvic pain, dyspareunia (positional or permanent), dyschezia, and dysuria.

There are many other pain presentations that nobody even thinks of until confronted with an endometriosis patient who, incidentally, has exactly “that type of pain”. A young girl we operated last year referred to right shoulder pain at menstruation. At laparoscopy, a large diaphragmatic series of blue and red lesions was excised. She was relieved after surgery.

A similar case was reported recently by Singh *et al*. ^27^. This publication elucidates the use of MRI for the clinical diagnosis of endometriosis, which will be shown extensively in this review.

Involuntary infertility, even when not the cause for consultation, should also be regarded as one of the frequent symptoms of endometriosis. Less frequently, cyclic nasal bleeding, umbilical bleeding, cyclic hemoptysis, cyclic constipation, and urinary urgency are reported by patients with endometriosis.

### B. Pelvic examination

Even today, with the advancement of imaging diagnosis, pelvic examination (in expert hands) continues to be praised as an effective clinical tool for the diagnosis of endometriosis. It should be done with care, slowly, beginning with abdominal palpation. Only after no pain is registered, proceed to pelvic examination. This should be done with extreme delicacy and respect. Bimanual palpation of the uterine/bladder pouch, the Douglas pouch, and adnexa can reveal exquisitely painful sites typical of endometriosis.

Fixed uterine retroversion is frequently due to uterosacral ligament compromise or adhesions at the Douglas pouch. Painful uterine mobilization is another typical sign of endometriosis. Compression of the uterine fundus is frequently painful when adenomyosis is present. Dyspareunia frequently corresponds with extremely painful palpation of the uterine-sacral ligaments.

Always look at your patient’s face during examination. Rictus of pain cannot be avoided. It will tell you exactly where the pain is more intense, helping to clinically determine the extent of the disease. Careful and expert pelvic examination provides a lot of information at a very low cost.

### C. Biomarkers

As of today, of the many biomarkers for endometriosis proposed in peripheral blood and endometrium, not one has been validated for endometriosis ^18^. This could be due to patient selection, sample collection, or analytical procedures. There is a current need to develop a non-invasive test for patients with symptomatic endometriosis.

We still lack a reliable marker for the disease. Ca 125, considered a marker for endometriosis, is helpful only in postoperative follow-up. It usually decreases after surgery and rises when the disease recurs or progresses.

Clinical presentations vary. Signs, symptoms, and markers do not correlate well with the extent of disease, as stated by Taylor *et al*. ^19^. In 58 consecutive cases of endometriosis, Hirsch *et al*. ^28^ found increased values of Ca 125. This group concluded that Ca 125 of at least 30 units per milliliter is “highly predictive of endometriosis” in symptomatic patients ^19^. The authors propose it as mandatory but consider it “unable to rule out endometriosis” ^19^.

Many publications describe gene abnormalities in patients with endometriosis. It would take a whole chapter to name them but none has yet been validated for the diagnosis of endometriosis. These alterations have been reported for the last 15 to 20 years. Some are showing ties with the disease. The large number of different approaches shows that the road is still unclear.

In 2016, after a systematic search of the literature, Neil Johnson, Cyndy Farquhar, and the Cochrane Library group found only two biomarkers—PGP 9.5 (neural fiber marker) and CYP19 (hormonal marker)—that showed enough accuracy to replace surgical diagnosis ^29^. Even so, the authors state that “we could not statistically evaluate most of the biomarkers assessed in this review in a meaningful way. In view of the low quality of most of the included studies, the findings of this review should be interpreted with caution. Although PGP 9.5 met the criteria for a replacement test, it demonstrated considerable inter study heterogeneity in diagnostic estimates, the source of which could not be determined” ^29^.

Blood, urinary, and endometrial markers—alone or combined with imaging—were analyzed. The authors conclude that none could be evaluated in a meaningful way. For them, there was insufficient or poor-quality evidence. There is a clear final recommendation: “Laparoscopy remains the gold standard for the diagnosis of endometriosis and using any non-invasive tests should only be undertaken in a research setting” ^29^.

### D. Genetics

For many years, there has been a search for genetic testing that could identify a population prone to develop endometriosis. A simple literature search identifies more than 3000 publications from 2018 linking genetics to endometriosis. Recently, an Australian group presented a summary of 17,045 cases included in a meta-analysis ^30^. In them, 14 genomic regions were identified, supported by results from multiple studies. The group found that “no independent associations were identified from direct genotyping of common and low-frequency protein-coding variants” ^30^. According to them, the most common genetic factors related to endometriosis risk are located in regulatory DNA sequences. This, they say, alters the regulation of gene transcription. They conclude that the target genes are present in three chromosome regions: “LINC00339 and CDC42 on chromosome 1, CDKN2A-AS1 on chromosome 9, and VEZT on chromosome 12” ^30^.

Using single-nucleotide polymorphism (SNP) array technology, a 2017 publication ^31^ describes genomic aberrations linked to the development of endometriosis. These investigators performed SNP array genotyping of pooled DNA samples from 100 patients with endometriosis and 50 controls. The authors detected 49 copy number variation (CNV) loci that were present in patients with endometriosis but that were absent in the control group. Six novel CNV loci in the subtelomeric regions representing gains and losses were identified. An intergenic locus on chromosome 19q12.1 showed a strong association with endometriosis. As with other biomarkers, we still lack a reliable genetic marker for endometriosis, and none of the proposed genes or gene alterations can be used to make a precise diagnosis.

### E. Imaging

***Ultrasound.*** In 1979, Walsh *et al*. presented their findings in 25 patients with surgically confirmed endometriosis or adenomyosis or both ^32^. Sonolucent zones within the uterus representing blood lakes described adenomyosis. Other cases had cystic images, five of which were of mixed characteristics. At that time, “ultrasound alone could not differentiate endometriosis from diseases such as tubo-ovarian abscess, ruptured ectopic pregnancy, other ovarian cysts or tumors” ^32^. The authors stated that the clinical history contributed to the non-surgical diagnosis of endometriosis.

Today, some authors state that TVS “allows a better accurate diagnosis of rectosigmoid endometriosis than MRI” ^33^. For this group, it is less reliable in the case of uterine, Douglas pouch, and uterosacral ligament disease. Nevertheless, they propose it as a first-line imaging technique because of its low cost and feasibility.

The International Deep Endometriosis Analysis group ^34^, confronting the wide variety of terms and descriptions used to identify endometriosis at TVS, proposes some basic steps that should be followed at the time of examination:

1. Routine evaluation of uterus and adnexa (search for adenomyosis and presence, or absence, of endometriomas)
2. Evaluation of transvaginal sonographic soft markers such as specific tenderness and ovarian mobility
3. Assessment of the Douglas pouch status (sliding sign)
4. Assessment for DIE nodules at the anterior and posterior compartments.

All steps should be performed, though not necessarily in this order, with a small liquid content in the bladder. A dynamic examination assessing the real-time mobility of the pelvic organs is mandatory in these cases.

This article includes a series of drawings and photos that accurately describe the different images related to endometriosis in all of its presentations. For those who practice the steps mentioned above, TVS is the first-line investigative tool in patients with symptoms of endometriosis. The ability demonstrated by them to detect ovarian endometriomas and DIE is well documented. The prediction of the pouch of Douglas obliteration is very accurate. It helps to organize multidisciplinary surgical teams in the most severe cases. They give most importance to the sliding sign since it allows clinicians to predict the severity of the deep pelvic disease. One possible drawback is the issue of experience: only those who have performed more than 2500 scans can achieve real proficiency in the sliding maneuver, after about 40 examinations. Any trained staff can manage this non-invasive diagnostic method for other locations of DIE except for rectovaginal septum DIE. A plight for a standardized nomenclature of US findings is mandatory for this group.

Another group presented clear and sound images of DIE in a prospective study ^35^. They evaluated the wall of the rectum and the lower sigmoid colon with two consecutive TVSs. The first was performed without previous bowel preparation, and the second after a 3-day low-residue diet and two 250-mL enemas (12 and 3 hours before TVS). They demonstrated that TVS after bowel preparation had a higher accuracy, allowing the detection of DIE before surgery.

Transvaginal US is the first option for the imaging diagnosis of ovarian endometriomas. A 2002 meta-analysis performed by Moore and Kennedy *et al*. ^36^, reviewing seven articles that fulfilled the inclusion criteria, demonstrated that TVS is a useful test in the case of ovarian endometrioma.

In a retrospective observational study, an Italian group ^37^ used TVS to evaluate 250 women of reproductive age (20 to 40 years) presenting endometriomas larger than 20 mm in diameter. The mean endometrioma diameter was 40 mm. Bilateral disease was found in 25.5% of the patients, posterior rectal DIE in 21.5%, and the thickening of at least one uterosacral ligament in 35.4%. Seventy-three percent of the patients showed adhesion signs, and 53% had concurrent uterine adenomyosis.

Only 15% of the studied population presented a single isolated endometrioma with a mobile ovary and no other signs of further disease. This publication highlights the utility of TVS for the diagnosis of endometriomas, adjacent coexisting disease in other locations, and adherences. In 50 patients operated by laparoscopy, TVS diagnosis was confirmed.

In 85% of the cases, endometriomas were associated with other locations of endometriosis. Left-side cysts were more frequently associated with same-side uterosacral ligament infiltration and DIE. Bilateral endometriomas usually obliterate the pouch of Douglas.

***Computerized axial tomography.*** “Computed tomography has no role in the routine evaluation of endometriosis except in very few particular scenarios” ^38^. An inguinal endometriotic nodule and a case of round ligament endometriosis that looked like a hernia were the only references found after a quick search of different databases, including Medline, linking endometriosis and computerized axial tomography (CAT) scans. Contrast studies might be of use for the diagnosis of ureteral stops, stenosis, or deviations in the case of lateral pelvic side-wall DIE.

CAT virtual colonoscopy can also be of help. A recent study describes its use before surgery for DIE ^39^. Associated with MRI, the preoperative diagnosis was confirmed in 71 patients who presented a total of 105 endometriotic bowel lesions. This group found 71.2% rectal nodules and 60% sigmoid nodules that infiltrated the muscularis propria in extensions varying from 25% to 50% of the circumference. Stenosis was present in 73% to 96% of the cases.

“The concordance between intraoperative and preoperative findings concerning the presence of rectal nodules was high, at 0.88 when associating CTC [computed tomography of chemiluminescence] with MRI, whereas each imaging technique taken individually provided lower concordance coefficients” ^39^. In this study, 80.3% of patients underwent the procedure that had been preoperatively planned. These authors propose that the association of both techniques improves the accuracy of preoperative assessment of colorectal DIE.

***Magnetic resonance imaging.*** In 1999, a pioneer article described the use of MRI for the preoperative diagnosis of endometriosis ^40^. The authors described, in 20 patients, MRI findings of DIE at the uterosacral ligaments, the pouch of Douglas, the rectum, and the bladder that were histologically proven at surgery. Diagnosis was accurate except when contrast was not used (two of three patients with rectal endometriosis).

A decade before, Arrivé *et al*. ^41^ published the first report of MRI use for the clinical diagnosis of endometriosis. Using only 0.35 Tesla, they prospectively studied 30 consecutive women with symptomatic disease. In 25 cases, endometriosis was confirmed by surgery. A sensitivity of 64% and a specificity of 60%, with an accuracy of 63%, were shown. Most endometriomas were correctly identified. Only 14 of 29 cases of adhesions and 6 of 45 cases of peritoneal implants were diagnosed by MRI. “MRI findings did not correlate with the surgically determined severity of the disease” ^41^. In 1989, the authors concluded that MRI could not be used as the first study to detect endometriosis. For them, laparoscopy was the procedure of choice.

**Figure 1.**
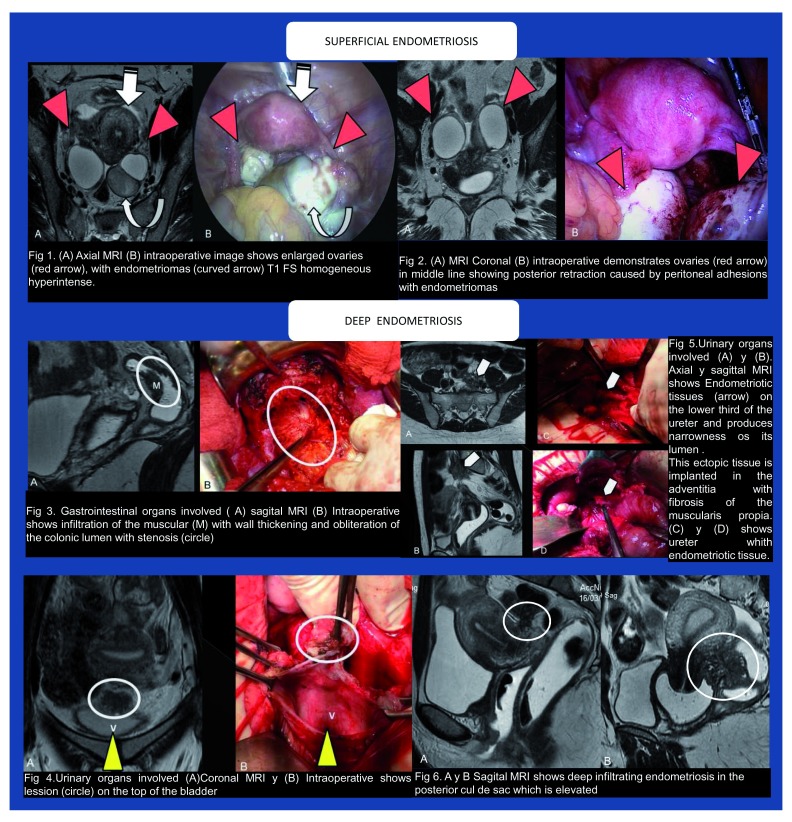
High-resolution magnetic resonance imaging and laparoscopic findings.

Last year, at the XIII World Congress on Endometriosis, our group presented three posters ^42– 44^ that established a perfect correlation of high-resolution contrast MRIs with laparoscopic findings, using 2- and 3-Tesla devices. MRIs and intraoperative images from one of the posters are displayed in Figure 1.

Using a high-resolution technique (1-mm slices), intravenous contrast (for bladder visualization), and vaginal and rectal gel contrast (for better visualization of the rectovaginal septum), we were able to stage the disease before laparoscopy. We demonstrated the special ability of this method to visualize superficial implants, adhesions, uterosacral ligament infiltration, rectovaginal septum infiltration (including the depth of rectal invasion), bladder wall infiltration, and ovarian disease. Images of ureteral compromise were also obtained (unpublished). Whenever possible, MRI would be mandatory before laparoscopy.

A recent publication ^45^ shows an interesting algorithm that allows clinicians to predict the probability of bowel resection at the time of laparoscopy for DIE using MRI. In 52 patients studied preoperatively, a positive predictive value of 87% and a negative predictive value of 83% were demonstrated. This group calculated the impact angle and lesion size by using a mathematical algorithm.

In 2009, the PRISMA (Preferred Reporting Items of Systematic reviews and Meta-Analyses) group proposed an evidence-based minimum set of items required for reporting in systematic reviews ( http://www.prisma-statement.org). A recent publication ^46^ evaluated the use of TVS and MRI for the diagnosis of adenomyosis, reviewing evidence in accordance with PRISMA requirements.

For TVS, they found a high heterogeneity between studies. The pooled positive likelihood ratios for adenomyosis were 0.72–0.82, 0.85–0.81, and 4.67–3.7, a clearly diffuse variety of information. In contrast, the pooled sensitivity for MRI was 0.77, the specificity 0.89, the positive likelihood ratio 6.5, and the negative likelihood ratio 0.2 for all subtypes of adenomyosis and publications. This suggests that MRI is more useful than TVS for the diagnosis of adenomyosis.

## Laparoscopy

Laparoscopy is the “gold standard” for the diagnosis of endometriosis. It certifies the presence of the disease and its extension. By means of tissue biopsies and its pathological analysis, the aggressiveness of the lesions can be determined. It is also the opportunity to perform the initial treatment of endometriosis, as will be described later in this article.

## Classifications

The WES ^47^ consensus on the Classification of Endometriosis was held at the XII World Congress on Endometriosis in São Paulo, Brazil, in 2014. Fifty-five representatives of 29 national and international, medical and non-medical organizations from a range of disciplines contributed.

It produced a statement that says: “until better classification systems are developed, we propose a classification toolbox” ^47^. This includes the revised American Society for Reproductive Medicine (rASRM) classification, the Enzian classification, and the endometriosis fertility index (EFI).

The most used staging system is the rASRM classification (1997), which ignores DIE. Kecktein in 2003 and Haas in 2013 proposed the Enzian classification for DIE as a complement to rASRM. In 2010, Adamson and Pasta introduced the EFI, although it is strictly related to endometriosis-associated infertility.

The Consensus reported that “however, the classification systems in current use continue to attract criticism from women with endometriosis and those providing care for them because of the poor correlation with disease symptoms as well as a lack of predictive prognosis and, to date, unclear pathways of treating pelvic pain and infertility based” on them ^47^.

### Revised American Society for Reproductive Medicine classification

**Figure 2.**
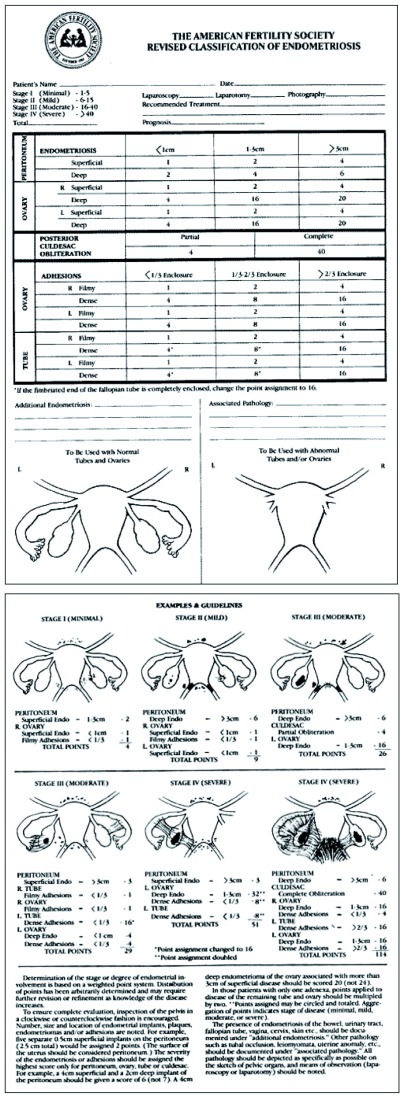
American Society for Reproductive Medicine revised classification.

In Figure 2, the rASRM classification is deployed. It was originally thought of by Acosta *et al*. ^48^ in 1973 while working with V. C. Buttram and after analyzing the results of 107 infertile patients operated for endometriosis. This group designed a simple scheme that evolved into the first ASRM classification. This classification is intended mainly to be used for those endometriosis patients consulting for infertility. DIE is not considered in this scheme.

### Enzian

**Figure 3.**
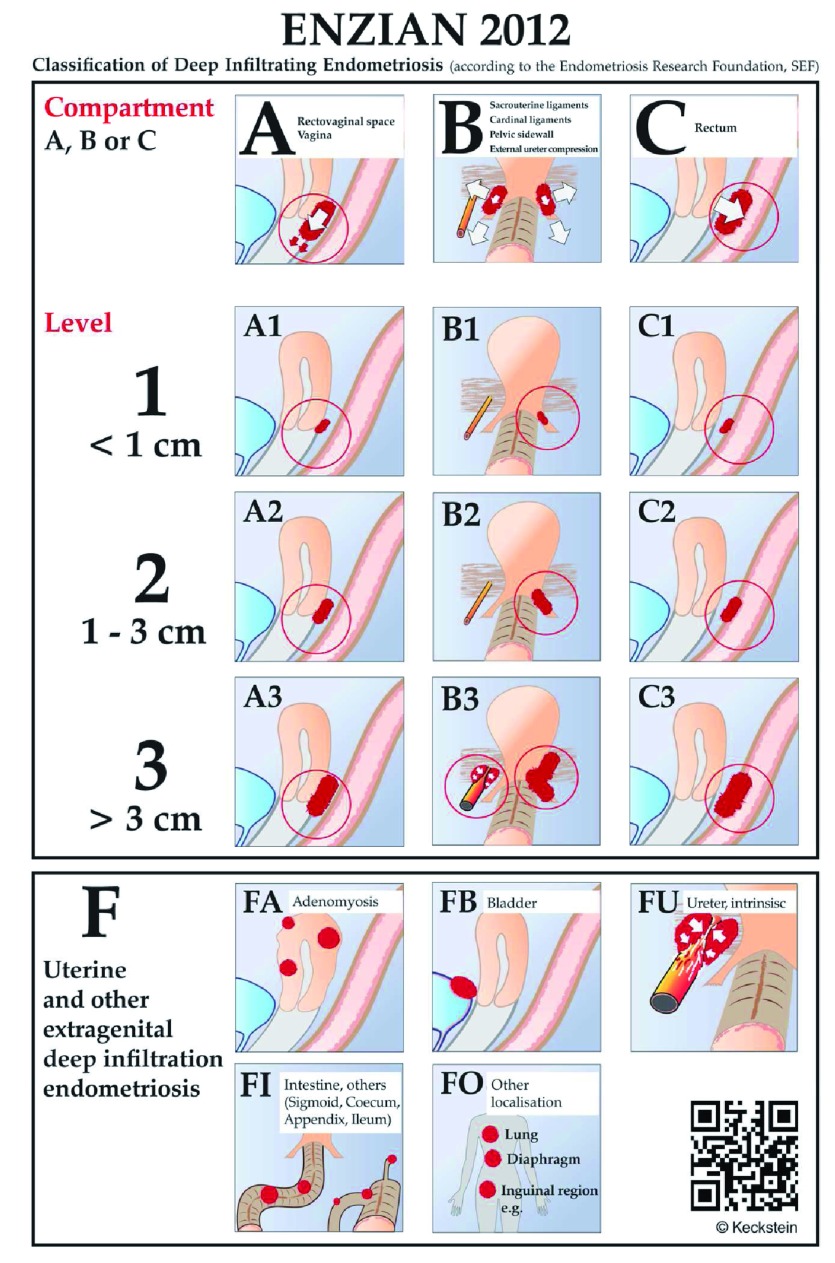
The Enzian classification for deep infiltrating endometriosis ^51– 53^.

The Enzian proposal, which includes DIE, is shown in Figure 3. Clear drawings help the surgeon to better stage the disease. This classification addresses the issues of the posterior compartment DIE and bladder (anterior) and ureteral (lateral) compartments.

### Fertility index

**Figure 4.**
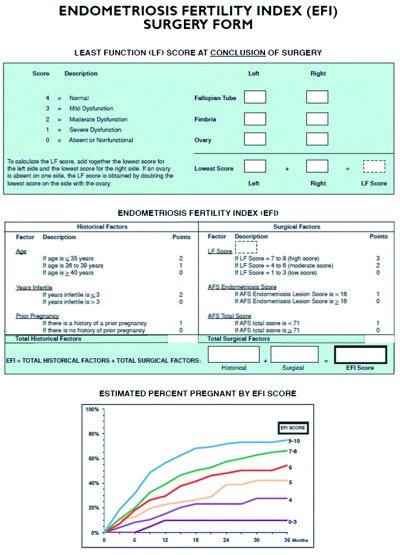
Endometriosis fertility index.

Adamson considered the poor fertility prognosis derived from the exclusive use of the ASRM modified classification. In 2010, he introduced the EFI, shown in Figure 4 ^49^, as a complement that allows a better diagnosis of the endometriosis-associated fertility status. Validated by several other authors, such as Hobo *et al*. ^50^, this index includes not only the laparoscopic findings (least functional score at the end of surgery) but also other issues that affect fertility, such as the length of infertility, patient’s age, and previous pregnancies.

### Drawbacks

A major drawback of all existing classifications is that no one of them links the severity of the pain with the findings. Some patients who would be classified as “severe” by ASRM revised charts experience little pain but do not get pregnant. Others, with only superficial red and blue lesions and minor adhesions, experience severe pain and a poor QOL. They probably would be entered as “mild” or even “minimal” using this proposal. The same occurs with DIE and pain. The question is: Should we include pain as a new item when establishing a definitive classification? Adamson, in a recent update on classifications ^49^, reports that the American Association of Gynecological Laparoscopists is developing a categorization system that will be more focused on pain.

### Koninckx and Wattiez *et al*.

In 2011, Koninckx and Wattiez *et al*. ^54^ presented a proposal that included adenomyosis, peritoneal pocket lesions, and subtle endometriosis plus the three traditionally recorded lesions (peritoneal, cystic, and deep). It considers the size of each lesion and includes pain as an issue. They state that the significance of the subtler lesions is not clear since they have not “been demonstrated to be associated with pain or infertility” ^54^.

**Figure 5.**
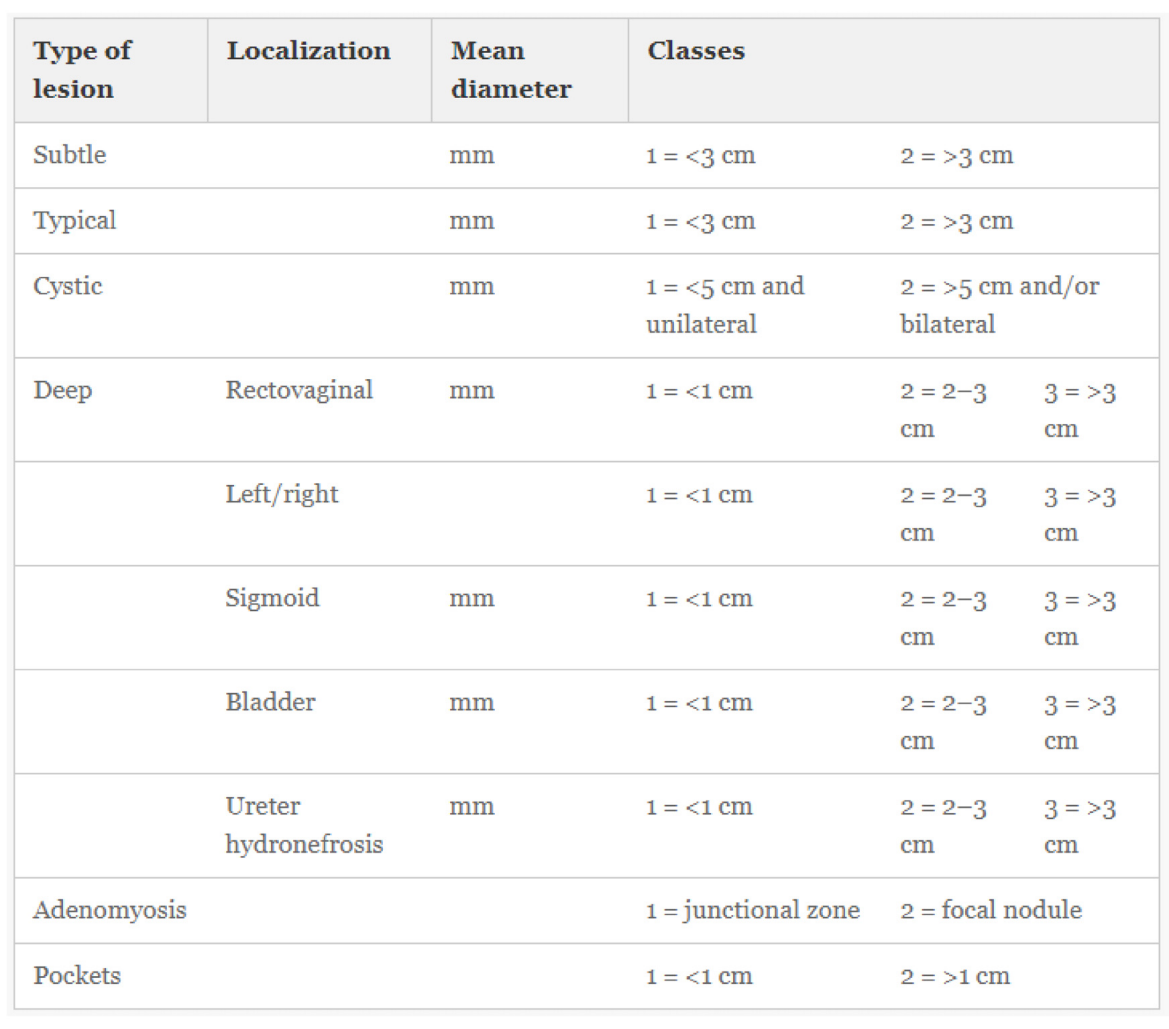
A new proposal by Koninckx and Wattiez *et al*. ^54^.

For them, subtle lesions and DIE (any lesion deeper than 5 mm under the peritoneal surface) should be classified apart. In regard to pain, they cite authors who link pelvic pain with lymph node involvement in the case of DIE. That is, lymph compromise is a marker of more intense pain. In Figure 5, we depict their classification proposal, which is not yet validated.

### Other proposals

In 2011, Charles Miller and Mauricio Abrao presented an article entitled “Six Good Reasons for a NEW Endometriosis Classification” ^55^. They propose that a classification should do the following:

1. Clearly describe the sites and extent of disease—peritoneal, ovarian, and deep endometriosis, including bowel, ureter, and bladder.
2. Provide a close correlation with the symptoms of endometriosis: pain (dysmenorrhea, dyspareunia, dysuria, and dyschezia) and infertility.
3. Reflect the surgical difficulty encountered relative to the disease location. This is facilitated by the inclusion of increasing radical procedures such as ureterolysis and bowel resection.
4. Be user-friendly with tools that are conducive to support a surgeon’s busy practice by enabling completion of documentation immediately upon procedure conclusion.
5. Be validated for both pain and infertility. With proper validation, this new system can be most useful for therapeutic and prognostic considerations.
6. Create a comprehensive universal language that is meaningful for clinical practitioners and researchers alike, thus providing the foundation of collegial collaboration, which ultimately will advance our understanding of the disease.

These concepts should be included if (ever) a single, global classification is finally created.

## Management

### Overview

Vercellini and Somigliana *et al*. summarize current available medical treatments for symptomatic endometriosis in a recent publication ^56^. According to them, they all act by inhibiting ovulation, reducing serum estradiol levels, and diminishing uterine blood flow. They state that several drugs can be used “with a similar magnitude of effect, in terms of pain relief” ^56^. They can be categorized by price, as low-cost drugs (OCs and most progestogens) or high-cost drugs (dienogest, GnRH agonists, and the newly introduced elagolix). They recommend starting treatment with low-cost drugs and step up to high-cost ones only in case of “inefficacy or intolerance” ^56^. For them, OCs are useful for superficial peritoneal disease or endometriomas smaller than 5 cm in diameter, and progestogens have a better effect in severe dyspareunia associated with deeper infiltrating lesions.

Back in the late 1970s, danazol NR was “the” drug. It relieved pain after diagnostic laparoscopy or conventional ovarian cystectomy—not much more was accomplished in the operating room (OR) at that time. Patients gained weight, grew a beard, and had elevated hepatic enzymes, but pain was gone, and progression probably arrested.

In 1988, Taymor *et al*. ^57^ questioned its clinical efficacy in infertile women. This prospective randomized study showed danazol to be ineffective in improving pregnancy rates over doing nothing at all in patients with minimal endometriosis. Years later, the World Consensus for the Current Management of Endometriosis ^11^ recommended it only before IVF in severe endometriosis.

Danazol is a drug of the past. OCs, progestogens, GnRH agonists, and lately GnRH antagonists (elagolix) and (to some extent) hormonal receptor modulators (such as ulipristal) are current specific medications. The role of antiestrogens is not clear, nor is that of natural origin substances (such as resveratrol), anti-aromatases, anti-angiogenic molecules, and immunomodulators.

### Pain

Pain is the most common reason for consultation; moreover, infertile patients often have chronic pelvic pain as well. Arrest of pain is always a priority.

We presented a poster at the XI World Congress on Endometriosis (Montpellier, France, 2011) and posters at the XII World Congress on Endometriosis (São Paulo, Brazil, 2014) showing the immediate pain relief and positive changes in over 100 studied patients. Their QOL improved immediately after a correct laparoscopic surgery in superficial endometriosis of different severities.

Two surgeons—Jose M. Curto and myself—participated. The quality of surgery was standard and reproducible. Surprisingly, 100% of the patients indicated an immediately better QOL after surgery. In all cases, postoperative treatment was allocated to control pain or infertility or both.

The traditional management of pain includes different approaches and different pathways. The group headed by Catherine Allaire states that, other than non-steroidal anti-inflammatory drugs (NSAIDs), “further work is required for nonhormonal therapies such as antiangiogenic and immune-modulating drugs” ^58^. They list as “traditional hormonal therapies” estrogen-progestin contraceptives, progestins, and GnRH agonists. Other hormonal treatment options are androgens and aromatase inhibitors. “Research also suggests a possible role for GnRH antagonists and selective progesterone receptor modulators, always discussing with each patient side effects and/or desire for pregnancy to ensure personalized treatment and optimal outcomes” ^58^.

Curiosities still occur in this field, such as the method used by George *et al*. ^59^ to treat persistent endometriosis chronic pain in a 32-year-old patient. That group administered 10 daily, 20-min sessions of 2-mA anodal transcranial direct current stimulation over the left primary motor cortex. In their experiment, visual analog scale pain symptoms were reduced by 60%, and at the 4-month follow-up, the patient still expressed an overall decrease in pain symptoms of 30%.

The European Society for Human Reproduction and Embryology (ESHRE) ^60^ proposes a clear and simple guideline. It recommends, as a Good Practice Point, to “counsel women with symptoms presumed to be due to endometriosis thoroughly, and to empirically treat them with adequate analgesia, combined hormonal contraceptives or Progestagens” ^60^. It nevertheless states that “it is clearly a paradox that by recommending empirical treatment in symptomatic (young) women” we might induce a delay in diagnosing the disease ^60^.

***Hormones.*** The guideline recommends prescribing hormonal treatment—combined OCs, progestagens, anti-progestogens, or GnRH agonists—“as one of the options, as it reduces endometriosis-associated pain” ^60^. It also “recommends that clinicians take patient preferences, side effects, efficacy, costs and availability into consideration when choosing hormonal treatment for endometriosis-associated pain” ^60^.

Combined estrogen and progestin OCs are recommended, as they reduce endometriosis-associated dyspareunia, dysmenorrhea, and non-menstrual pain, in a continuous protocol. Also, vaginal contraceptive rings or transdermal estrogen/progestin patches are suggested.

Recommended progestogens are medroxyprogesterone acetate (oral or depot), dienogest, cyproterone acetate, and noretisterone acetate. Anti-progestogens such as gestrinone are considered. The different side effect profiles of each one of those drugs, especially thrombosis and androgenism, should be regarded. The use of a levonorgestrel-releasing intrauterine device is also an option. Aside from difficult cycle control in some users, dienogest in doses of 2 mg per day results in very effective pain reduction and control of the disease progression. It can be used over prolonged periods of time provided that no recurrent bleeding occurs. This is the main cause of discontinuation.

***Anti-hormones.*** According to the guideline, evidence regarding dosage or duration of GnRH agonists is limited. “Clinicians are recommended to prescribe hormonal add-back therapy to coincide with the start of GnRH agonist therapy, to prevent bone loss and hypoestrogenic symptoms during treatment” since this will not reduce the effect of the pain treatment ^60^. The guidelines recommend giving “careful consideration to the use of GnRH agonists in young women and adolescents, since these women may not have reached maximum bone density” ^60^.

***Aromatase inhibitors.*** These options are considered for those who have pain from rectovaginal endometriosis, refractory to other medical or surgical treatment. They should be prescribed in combination with OC pills, progestagens, or GnRH analogs.

***Analgesics.*** The guideline asks a simple question ^60^: “Are analgesics effective for symptomatic relief of pain associated with endometriosis?” Then it tells us that evidence on the use of NSAIDs for endometriosis is scarce. The authors of the guidelines present a referenced publication from 1985 and one study on the cyclooxygenase-2 (COX-2) inhibitor rofecoxib (withdrawn from the market in many countries because of severe side effects). For the guideline, NSAIDs have a “favorable effect on primary dysmenorrhea and are widely used as a first-line treatment of endometriosis-associated pain” ^60^. However, it recommends the use of NSAIDs or other analgesics to reduce endometriosis-associated pain. Clinicians should discuss the associated side effects with the patient.

The off-label use of a COX-2 inhibitor, etoricoxib, available in many countries could replace rofecoxib. As was demonstrated long ago ^61^, its tolerance and fewer upper gastrointestinal clinical events versus the traditional NSAID diclofenac are notable, although this difference was not reported in relation to the rare and very serious adverse side effects that both drugs can produce. Petraglia *et al*. ^62^ studied rofecoxib back in 2004, before it was banned, in 16 patients versus 12 in a placebo group in a dose of 25 mg per day for 6 months. In this group of young patients, no significant side effects occurred. Pain relief was significant and persistent, making this a “safe and low-cost therapy for endometriosis associated pain” ^62^. A future preclinical randomized controlled trial (RCT) is needed to replace COX-2 inhibitors in the public awareness as an option, using alternative drugs such as the previously suggested etoricoxib. The Practice Committee of the ASRM states that “first-line medical treatment for pain due to endometriosis is often a nonsteroidal anti-inflammatory drug” ^63^. This everyday practice, difficult to change, affects women without a certified diagnosis of endometriosis.

The Cochrane Library ^64^ recently reviewed the use of NSAIDs for endometriosis-associated pain. The Cochrane Gynecology and Fertility Group Specialized Register of Controlled Trials (October 2016), MEDLINE (January 2008 to October 2016), and the Embase project (January 2016 to October 19, 2016) were analyzed. All RCTs describing the use of NSAIDs for the management of endometriosis-associated pain in women of all ages were included. The available evidence was very low, measured by the GRADE system (Grades of Recommendation Assessment, Development and Evaluation, a public domain system that describes evidence as “very low” when the true effect is probably very different from the estimated effect, “low” when the true effect might be very different, “moderate” if the true effect is probably close to the estimated effect, and “high” when the true effect is similar to the estimated effect). Comparison of NSAIDs (naproxen) versus placebo revealed no evidence of a positive effect on pain relief, although women taking NSAIDs (naproxen) were less likely to require additional analgesia. Those studies provided no data on QOL, effects on daily activities, absence from work or school, need for more invasive treatment, or participant satisfaction with treatment. No judgment as to whether NSAIDs (naproxen) are effective in managing pain caused by endometriosis can be made. There is no evidence that one NSAID is more effective than another. As shown in other Cochrane reviews, women taking NSAIDs must be aware that these drugs may cause unintended effects.

Opioid derivatives such as codeine and tramadol have rarely been studied for the treatment of endometriosis-associated pain. Many doctors prescribe them to these women ^65^: “Obstetrician–gynecologists reported prescribing a median of 26 opioid pills across all indications combined”. “Opioid-related deaths recently exceeded motor vehicle accidents as the leading cause of injury-related death in the United States”. “In 2015, 2 million Americans had a prescription opioid use disorder, and more than half of those who reported prescription opioid misuse obtained the drugs through diversion of prescribed medications”. The use of this type of analgesics should be limited to very exceptional cases in which other drugs have failed and during the least span of time.

## What’s new?

### Elagolix

The elagolix phase III clinical trial ^66^ introduced a new and promising treatment for endometriosis. Oral anti-gonadotrophic agents have a sound future. They arrest the progression of the disease and dramatically reduce pain. Relief is fast and significant. New clinical trials for similar drugs are ongoing. In the publication of reference, elagolix compared with placebo showed a significant decrease from baseline in the mean pain score. This significant effect was seen at months 3 and 6 of treatment.

One adverse side effect, bone density damage ^67^, was dose-dependent. Decrease in lumbar spine bone mineral density (BMD) following 6 months of treatment with elagolix compared with placebo was significantly different for each elagolix dose. “The proportion of participants with lumbar spine BMD decrease from baseline greater than 3% was also dose-dependent”, as was the case of decreases in BMD of the total hip and femoral neck ^68^.

Current protocols for similar drugs include addback hormonal therapy to minimize the effect on bone density. How long can the patient be treated with GnRH antagonists that produce medically induced menopause-like status? This is a question for the near future since trials provide only up to 12 months of treatments. With addback therapy, these drugs could probably be used for prolonged periods of time.

On July 24, 2018, the FDA approved elagolix for the management of moderate to severe pain associated with endometriosis.

Recent studies ^69, 70^ address other advantages: dyspareunia and pain reduction across a range of baseline characteristics have been shown to be consistent.

During the first 12 weeks of treatment with either leuprolide acetate (LA) or elagolix, subjects had lower estradiol (E2) levels compared with women who received placebo. Estradiol levels were lowest among LA-treated women. The results were placebo = 0 (0%) reduction, elagolix 150 mg = 0 (0%), elagolix 250 mg = 6 (14%), and LA = 29 (67%). This indicates a need for lower hormonal addback doses, or none, depending on the dose administered.

### Proellex

Selective progesterone receptor modulators are a class of drugs with progesterone antagonist activity that may confer therapeutic benefit for reproductive disorders in premenopausal women. Endometrial structure, which is dynamically controlled by circulating sex hormones, is likely to be perturbed by progesterone receptor modulators through their progesterone antagonist properties.

A selective progesterone receptor modulator, CDB-4124, telapristone acetate (Proellex), was clinically studied recently ^71^. Its performance as a treatment for endometriosis was proven. It is a drug with progesterone antagonist activity. A major concern was the alteration of the eutopic endometrium. The structure of this tissue, dynamically controlled by circulating sex hormones, is altered by progesterone receptor modulators through their progesterone antagonist properties. A group of pathologists examined endometrial histology in 58 premenopausal women whose endometriosis was treated with the progesterone receptor modulator CDB-4124 in daily oral doses of 12.5, 25, or 50 at 3 and 6 months’ follow-up ^72^. Most of the endometrial biopsies (103 of 174 biopsies) contained histologic changes that are not seen during normal menstrual cycles. The endometrium was generally inactive or atrophic and, less frequently, proliferative or secretory. Some presented anomalies, including formation of cystically dilated glands, and secretory changes coexisting with mitoses and apoptotic bodies. Increasing daily dose and duration made the cysts predominant and their lining inactive or atrophic. None of the patients who received CDB-4124 developed endometrial carcinoma or hyperplasia while on therapy.

Proellex has been questioned by the FDA because of liver enzyme increases shown by patients who received treatment in the endometriosis clinical trial. Clinical trials were halted and the company that developed the drug is now redirecting future studies to vaginal administration. A partial halt of the studies has not yet been reverted by the drug authority.

### Ulipristal

In his doctoral thesis, Simpson ^72^, after a 3-month treatment course with ulipristal acetate (Esmya) for endometriosis-associated pain, found a good clinical response in 56% of the studied cohort. Post-treatment histological and immunohistochemistry changes were correlated to changes in the macroscopic appearance of the disease and changes in symptom severity. In this descriptive observational cohort study, ulipristal acetate appears to offer an effective treatment for endometriosis with histological changes in the eutopic endometrium that should be carefully observed. “The safety of this compound remains to be elucidated but the results from this pilot study are encouraging and should prompt further exploration”, wrote Simpson ^72^.

A possible relationship with endometrial malignancies and severe liver damage reported excludes it from currently available endometriosis drugs. The FDA recently (August 2018) refused, once again, to authorize Esmya (brand name for ulipristal acetate) for human use. The drug had been preliminarily approved by the EMA in 2015. In May 2018, the EMA issued a warning about the rare occurrence of liver complications. In June 2018, the agency approved its use in the preoperative treatment of fibroids (that is, for a short period of time). There could be a possible role for ulipristal acetate in the management of endometriosis: a surgical pretreatment. In my opinion, long-time use is not guaranteed to be safe at the present time. We must keep in mind that endometriosis is a benign condition and care should be taken when prescribing medications with dangerous side effects, even if rare and infrequent.

In regard to hyperplasia, some authors explain it as a singular endometrial alteration induced by progesterone receptor modulators and not a real hyperplasia. This modification of the endometrial structure would regress after drug cessation.

### Resveratrol

Resveratrol, a natural drug derived from grape wine, induces apoptosis in endometrial stromal cells via the suppression of *survivin* expression. Ines Baranao and her group at IBYME–CONICET (Buenos Aires, Argentina) are working in animal experimentation with endometriosis surgical implants in rats, where they demonstrate the suppressive effect of resveratrol on the progression of the disease. Makabe *et al*. ^73^ describe how it enhances apoptosis in endometriotic stromal cells. There is a long journey yet to be accomplished but these very preliminary results are promising ^74^.

## Alternative treatments

The Montpellier Consensus ^11^ included the following: acupuncture, high-frequency transcutaneous electrical nerve stimulation, Chinese herbal medicine, vitamins B _1_ and B _6_, magnesium, tropical heat, spinal manipulation, and behavioral interventions. No sound evidence or high-grade studies support those therapies, but if they cause no damage or delay specific treatment, they can be considered supporting therapies. Cannabis has been shown to be only moderately effective for the relief of chronic pain and has potentially serious side effects (and there are no studies specifically addressing endometriosis).

There are not enough comprehensive publications that address, with high-grade evidence, the variety of uses of psychotherapy. For instance, group therapy, support or self-help groups, physical activities, and music or drama as co-therapies may be used to allow patients to socialize and overcome their misfortunes and frustrations.

## Lifestyle: diet and exercise

No interventions on lifestyle, exercise, or diet have demonstrated with an acceptable evidence grade that they can be of help to improve QOL. On the other hand, most patients believe in them and find them useful. Cognitive therapies and yoga are favorites. Only small retrospective observational studies suggest that exercise “might be effective in reducing dysmenorrhea” ^11^. Weight reduction or specific dietary interventions have no clearly demonstrated effects except for the fact that diet after surgery, with vitamins, minerals, salts, and lactic ferments, appears to be effective in pain reduction in comparison with hormonal treatment. There is a consensus that gluten-free diets improve symptoms in some women who have endometriosis and gastrointestinal complains.

## Fertility

The opportunity, quality, and extension of the first surgery are determinant when fertility is the issue. Milani and Cesana *et al*. ^75^ recently evaluated the reproductive prognosis during the first three years after conservative surgery. In a retrospective study, they surveyed 140 patients operated for endometriosis (with histological confirmation). With no other infertility factors, the pregnancy rate in a group of previously infertile patients was 53%: 48 spontaneous pregnancies and 10 with ARTs. Those patients who had not sought pregnancy before surgery were also followed and 71% of them became pregnant. Only three in a group of 31 required ART. In this series, the prognosis was not related to rASRM stage at surgery or the presence of uni- or bi-lateral endometriomas, tubal adhesions, or superficial lesions. The pregnancy rate inversely correlated to the pouch of Douglas obliteration (with significance, *P* = 0.05).

**Figure 6.**
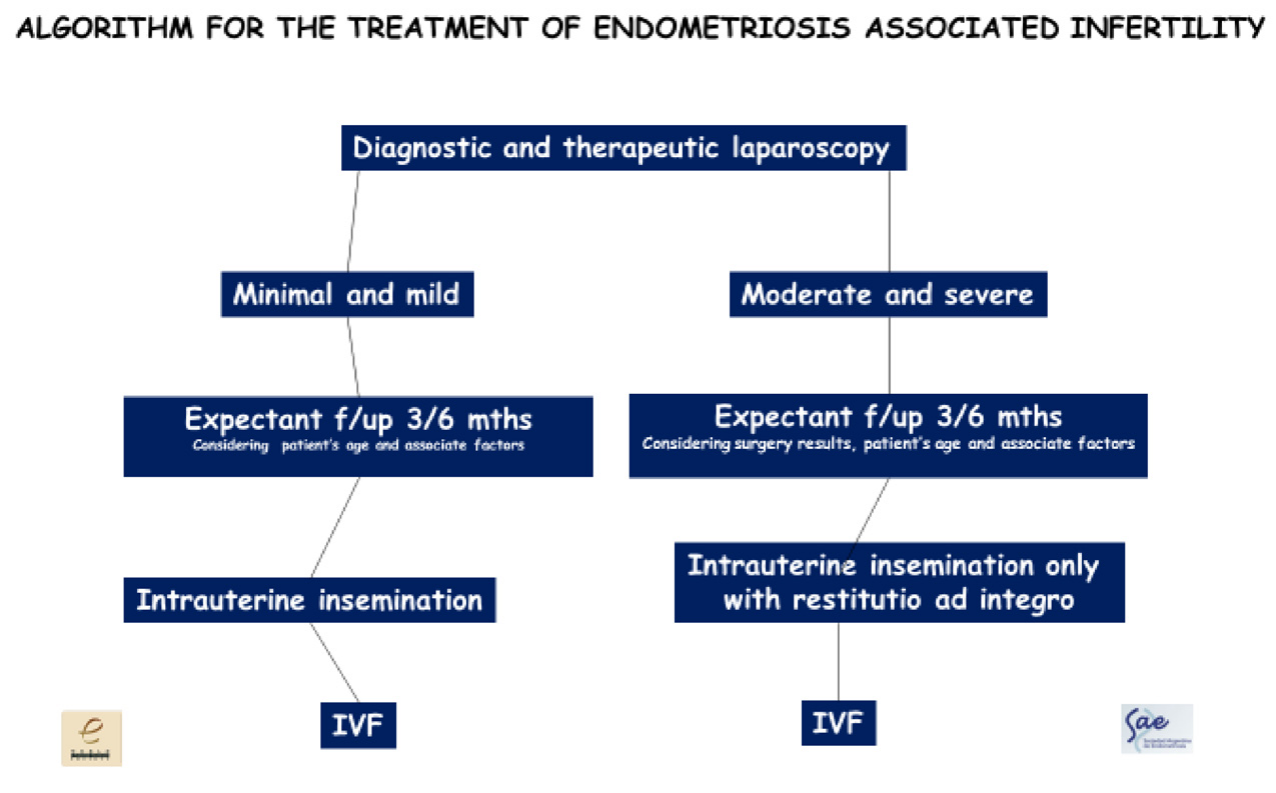
Algorithm proposed by the author for the treatment of endometriosis-associated infertility. This algorithm is proposed in cases where laparoscopy is indicated for endometriosis-associated infertility. Many patients with suspected endometriosis benefit directly from assisted reproductive techniques without a prior laparoscopy. IVF, *in vitro* fertilization.

I usually manage my patients by following this simple algorithm ^76^ ( Figure 6). This algorithm is proposed in cases where laparoscopy is indicated for endometriosis-associated infertility. Many patients with suspected endometriosis benefit directly from ARTs without a prior laparoscopy.

The World Consensus for the Current Management of Endometriosis ^11^ gives simple directions on how to manage infertile patients with endometriosis:

1. Principles of laparoscopic surgery for infertility are similar to those for other symptoms.
2. Surgical training and expertise are the keys for the best outcomes.
3. Ovarian reserve should be considered prior to surgery.
4. There is growing evidence that surgery of endometriomas affects ovarian reserve.
5. Pain is to be considered at the time to decide whether to proceed to surgery.
6. Surgery and ART should be considered complementary strategies.
7. Laparoscopic removal of endometriosis is effective to improve fertility in minimal and mild cases.
8. Lesion excision is preferred to thermal or laser destruction, especially in DIE where pain is an issue.
9. There is no high-grade evidence to assess whether surgery improves fertility in moderate and severe disease, including DIE.
10. Functional appearance of the tubes and ovaries at the end of surgery is related to the chances of natural conception afterwards.
11. Cystectomy for endometriomas larger than 4 cm in diameter, if possible, improves fertility more than simple ablation (drainage and coagulation).
12. Cystectomy should be performed with expertise and care, identifying tissue planes and carrying out careful dissection and avoiding the removal of surrounding ovarian tissue.
13. Suturing versus coagulation for hemostasis is better in order not to affect ovarian reserve.
14. Young patients should be counselled about oocyte cryopreservation prior to ovarian surgeries.
15. Observational studies suggest good fertility results after surgery for DIE.
16. Surgery for DIE should be considered as a second-line treatment after failed IVF.
17. Pregnancy rates after repeat surgery are low.
18. Two cycles of IVF might be more effective than second surgeries.
19. Surgery should be considered if pain is present or there are enlarging endometriomas as well as for those with repeated IVF failure or difficult access to such procedures.
20. Postoperative medical (hormonal) therapies delay and do not enhance pregnancy.
21. Except in the case of severe endometriosis before IVF.
22. Intrauterine insemination combined with ovarian stimulation is an effective option provided that tubes are patent.
23. The use of gonadotrophins appears to be more effective versus clomid.
24. IVF is first-line in preference in more severe cases, advanced female age, or reduced sperm quality.
25. Endometriosis may have a negative impact on IVF success rates.
26. It is mandatory if tubes are compromised.
27. IVF does not appear to increase the risk of recurrence of endometriosis.

For many authors, at IVF procedures after laparoscopic surgeries for advanced stages of the disease, the number of follicles recruited, access to ovaries at harvest, quality of oocytes, fertilization rates, and implantation success are severely compromised by endometriosis. These patients are difficult to treat by IVF, and repeated treatment cycles are the rule in endometriosis-associated infertility.

Implantation rates are compromised in adenomyosis as well. Laparoscopic surgical removal of as much as possible of the disease (including DIE) before IVF could enhance results. The resection of adenomyotic nodules, especially those located in the posterior uterine wall, when they measure 4 cm in diameter or more, would allow better results at the time of embryo transfer. This is a difficult and laborious surgery which requires delicacy and expertise. The uterine wall, as is the case in myomectomies, must be duly repaired in several overlapping planes to allow a normal developing pregnancy and reduce the incidence of uterine ruptures.

Younes and Tulandi ^77^ recently reviewed adenomyomectomy in reproductive-age women. Surgery should be offered when medical treatment is not suitable or effective, especially in the case of focal disease. In a review of 10 prospective and 17 retrospective studies comprising 1398 patients, the authors showed that excision is effective for symptom control (pain and hemorrhage) and probably also for infertility. Three fourths of those women seeking pregnancy conceived after surgery, with or without assisted reproductive adjuvant therapies. The best surgical procedure, for this author, is yet to be seen.

The possible mechanisms involved in endometriosis-associated infertility have not been completely elucidated ^78^. The oocyte is believed to have an important role. Oxidative stress events associated with alterations in the peritoneal, serum, or follicular microenvironments might result in poor oocyte quality. In this study, the possible mechanisms involved in oocyte quality impairment occurring in early disease are described. Another recent essay ^79^ indicates that oocyte quality is decreased in women with minimal or mild endometriosis.

Brugnon *et al*. ^80^ found that the number of collected oocytes, transfer rate, and the rate of cycles with a frozen embryo were lower in cases of endometriosis. Also, response to stimulation and good embryo quality cohorts were lower. But, for this group, the implantation, delivery rates, and cumulative live birth rates per attempt were like those of controls.

## Deep infiltrating endometriosis

Roman’s recent survey, which included 1135 patients from 56 different health-care centers in France, gives us an updated scenario of surgical treatment for DIE ^81^. More than half of the cases presented only rectal infiltration. In 36.3%, rectum and colon were affected. Sigmoidal-only lesions were less frequent (6.9%). Cecum, small bowel, and bladder involvement was even less frequent. In 13.4% of the patients, there was ureteral stenosis (6.8% associated with hydronephrosis). Most cases were operated by conventional laparoscopy (82.2%). Robotics and conventional surgery represented less than 10% of the total. Half of the patients were treated by rectal shaving and 40.4% by segmental resection of the rectum and sigmoid colon. Fistulas were rare, in both bowel and ureteral surgeries, and only one death was reported (after rectal shaving procedures). This study brings to the surface the relevance of DIE, which is far from being a rare disease. Interdisciplinary teams can achieve successful laparoscopic surgical treatments in most cases (9 out of 10).

Is there a place nowadays for medical treatment of DIE? Vercellini and Somigliana *et al*. ^82^ propose a challenging view of the importance of medical treatment in many cases of DIE. They begin their article with two controversial citations: (1) medical therapy for rectovaginal and colorectal endometriosis has been found to be ineffective or temporary (the rate of recurrence is as high as 76%) whereas surgical excision is effective in relieving pain (Minelli), and (2) one of the main characteristics of symptoms related to DIE lesions is that they dramatically respond to therapeutic amenorrhea (Fauconnier). Whom should we believe? “Clinicians and patients should know whether and to what extent medical treatments are effective for DIE, under what circumstances can they be used, and if they really constitute an acceptable alternative to surgical treatments” ^83^. The authors state that “different hormones, or hormonal combinations relieve pain and other symptoms of DIE. Improvement in dyspareunia, dyschezia, and bowel complaints suggests that they are efficacious” ^83^.

Few RCTs have been conducted for DIE medical treatments. Low-dose progestogens seem to be the best option and, for these authors, should be the first treatment option (norethindrone acetate and dienogest). GnRH with addback hormonal therapy could be used, even as a long-term option in selected cases with high surgical risk, but “conservative or definitive surgery” should be considered. For them, the “contraposition between medical and surgical treatment should be overcome by applying a stepwise approach” ^83^. Vaginal route could be advantageous in the case of rectovaginal lesions. The need to discontinue hormonal treatments when pregnancy is sought is an important reason to consider other options.

## Quality of surgery

Surgery should be provided by experts on the disease. Much of the recurrence (or persistence) of endometriosis is related to poor first surgery quality, incomplete removal of all lesions, or wrong attitude at the time of laparoscopy.

I want to stress the importance of laparoscopic surgery for the diagnosis and primary treatment of endometriosis. Certainty can be achieved only by laparoscopic staging and biopsy (for histological confirmation of the disease). Histology helps to make a better prognosis, according to the activity of the lesions. Laparoscopy gives the opportunity to excise all disease present, including adhesions, peritoneal lesions of all types (blue, red, white, scars, and peritoneal pockets), endometriomas, and deep infiltrating lesions. Preoperative workup, including contrast MRI, will allow the surgeon to correctly appraise each case before taking the patient to the OR.

The treatment of endometriosis requires a delicate and experienced surgeon and, if it is the case, an interdisciplinary team, including gastrointestinal surgeons or urologists (or both), in selected patients. Multidisciplinary pelvic surgeons may be available at some institutions, where a reduced number of gynecologists operate a large number of patients. In most environments, the number of surgeries per year required to sustain expertise in all areas of surgery might be impossible to achieve. Therefore, interdisciplinary surgery proves to be mandatory in most locations.

A recent online consensus meeting organized by the WES addressed the issue of “centers of excellence or expertise” in endometriosis and of “surgical experts” in DIE. In a preliminary presentation, many options and requirements were suggested to distinguish a surgeon as an “expert” in endometriosis. There is still a long way to go before a final consensus might be achieved. Surgical expertise by itself should not be a determinant: real interest in all aspects of endometriosis is required.

## Peritoneal lesions

Peritoneal lesions should be excised whenever and wherever possible. Many of them are, in fact, deep lesions hidden behind a typical blue, red, or white superficial appearance. The World Consensus for the Current Management of Endometriosis ^11^ gives the following recommendations: Although RCTs have failed to demonstrate beneﬁt of excision over ablation, excising lesions where possible is recommended, especially where pain is present (weak).

**Figure 7.**
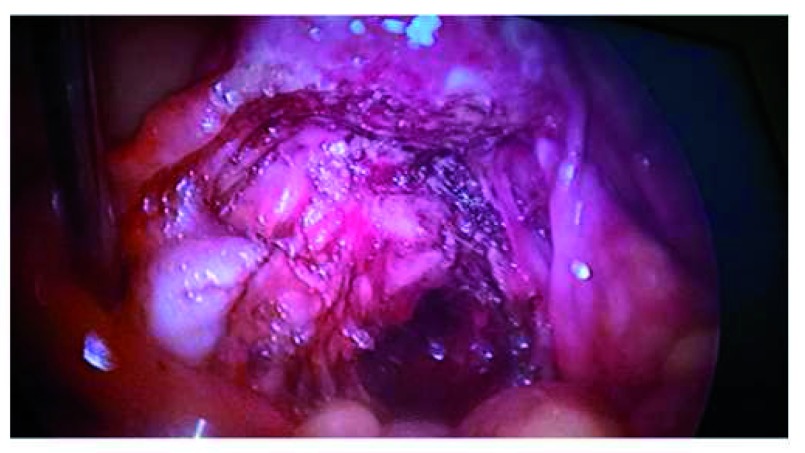
Complete removal of all superficial and hidden lesions at the pouch of Douglas. Image is from surgery performed by the author.

Although this might be disputed by many, destruction by electrocoagulation or laser does not allow histological study of the lesions, a reliable diagnosis, or the evaluation of the degree of functionality of the disease. In some cases, extensive “peritonectomies” must be performed in order to correctly remove all disease present ( Figure 7).

The ESHRE Guidelines clearly state that “at laparoscopy, deeply infiltrating endometriosis may have the appearance of minimal disease, resulting in an underestimation of disease severity. Evidence level 3” ^60^.

Taylor, Horne, and Adamson *et al*. ^83^ propose that “superficial endometriosis lesions present a particular diagnostic dilemma for physicians owing to their heterogeneous visual appearance and the fact that non-pigmented peritoneal lesions often represent highly active endometriotic implants”. This thought emphasizes the need for excision and histology proof if a correct diagnosis of endometriosis is our aim.

## Endometriomas

Somigliana ^84^, in a recent and very interesting brief article entitled “Ovarian reserve, endometriomas, and surgery: research must go on”, clearly synthetizes the current controversy on surgery for endometriomas. With a citation of Muzii, he states that the lower levels of anti-Müllerian hormone—considering it a marker of ovarian reserve—precede surgery, suggesting that the ovarian damage is due at least in part to the endometrioma itself and not only the surgical procedure.

The size of the endometriomas included in this study exceeds 5 cm in diameter, possibly indicating that the size counts and moreover that the larger the cyst, the greater the damage either to the ovary structure or to its function.

In any case, surgery for endometriomas is an issue that needs attention. Regularly, ovarian cysts are assigned by a pathology department to be operated by the less trained surgeons and, to the best of my knowledge, this is not the case with endometriomas. Needless to say, laparoscopy is the best surgical approach.

Stripping and removing the cysts, whenever possible, are considered the best options because they allow lower recurrence rates ^11^ and, in the case of infertility, better pregnancy rates. This is no easy task and requires expertise and delicate procedures. In my experience, the older cysts are more difficult to remove without damaging surrounding follicular tissue.

Again, the question of centers (and surgeons) of expertise arises. Surgery for endometriomas should be considered complex procedures to be carried out only by expert surgeons, especially in younger women who want to become pregnant.

The Working Group of the European Society for Gynaecological Endoscopy, the ESHRE, and the WES ^85^ make these recommendations:

1. Separate the ovary with endometrioma from the pelvic side wall, to which it usually adheres, by adhesiolisis. This usually results in drainage of the endometrioma. It is important to visualize the ureter at this stage to avoid damage, as the ovary may be adherent to it. In the presence of dense adherence, start the surgery by dissecting the ureter from the healthy tissue proximal to the adherence point. Endometriotic tissue on the pelvic side wall will need to be removed as well. (This will be covered in the subsequent recommendation on the treatment of peritoneal endometriosis.)
2. Where the cyst ruptures, extend the opening in the cyst wall adequately to expose the cyst cavity. Multiple incisions and excessive opening should be avoided to prevent damaging the ovarian cortex, functional ovarian tissue, and the hilum. Where feasible, the cyst may be turned inside-out to facilitate further treatment.
3. When the ovary is not adherent, the incision ideally should be over the thinnest part of the ovarian endometriotic surface or, if this is not visible, on the anti-mesenteric border.

Then a dissection plane should be identified, even if this requires cutting the side of the cyst until a good cleavage plane is seen. The injection of saline between planes could be of help at the time of the cystectomy. “Once the cleavage plane is identified, use gentle traction and counter-traction with appropriate instruments to dissect the cyst capsule from the ovarian parenchyma” ^86^.

**Figure 8.**
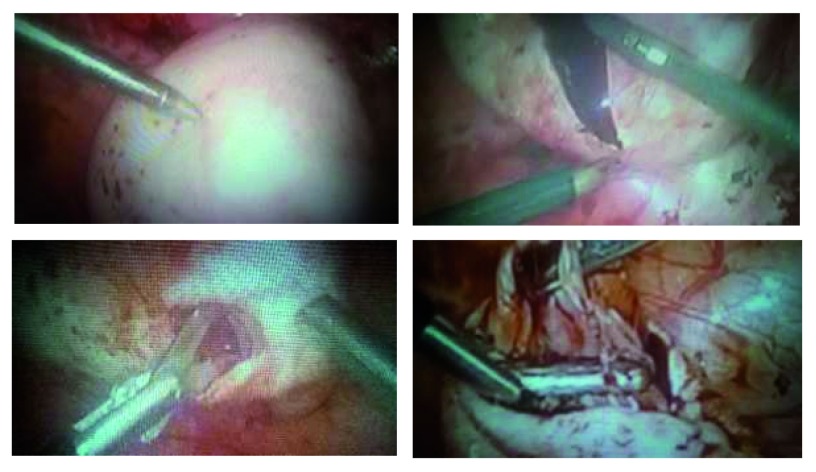
Endometrioma cystectomy. A correct dissection plane is mandatory for a careful cystectomy of endometriomas and must be clearly identified. Whenever possible, incision should be made at a site opposite the ovarian vascular pedicles. Image is from surgery performed by the author.

Only when total cystectomy is impossible, removal of as much of the thermal coagulation of the remaining portion as possible should be completed with laser. “Precise spot bipolar coagulation is the key to achieve hemostasis, to prevent unnecessary damage to healthy tissue”, according to the recommendations ^86^. That is, do a careful surgery avoiding blind coagulation of hemorrhagic tissue, which might result in over-coagulation of the ovarian ilium ( Figure 8).

The question of whether to close the surgical lodge with sutures is a matter of current controversy. We suggest that knots could aid hemostasis and avoid thermal damage and, in large endometriomas, give the remaining ovary a better shape for healing. Always use the thinnest fast re-absorbable sutures.

To operate or not to operate, that is the question. Current opinions differ depending on the size of the endometrioma, the age of the patient, the need for surgical staging and the treatment of other sites of the disease, the desire for pregnancy, and so on. Smaller endometriomas (that is, 2 cm in diameter) could be spared if IVF is a suitable procedure, but those larger than 4 cm ^11^ should be excised.

Ovarian reserve is more affected by recurrent surgeries in the same ovary. Thus, a delicate and complete cystectomy is the best procedure, as noted before.

Drainage and coagulation or laser destruction is not the best technique, since it also affects surrounding ovarian tissue and is linked to a higher recurrence (or persistence) rate.

The singer, not the song, say Muzii and Miller ^86^. For them, the quality of the surgery, and not surgery itself, is important. “Surgery is the gold standard treatment for ovarian endometriomas, but it should be performed with proper techniques by specifically trained surgeon” ^86^.

## Deep infiltrating endometriosis

A surgical chapter by itself, DIE remains the most complex surgical procedure in the domain of endometriosis. Four different scenarios are encompassed in DIE: bladder infiltration, lateral disease (with or without ureteral compromise), sigmoid affectation, and rectovaginal nodules. All of those surgeries, except for some minor bladder lesions, require expertise and, in many cases, interdisciplinary surgical teams, including gastrointestinal surgeons and urologists.

Bladder infiltration needs to be resected in total, even if the bladder must be opened and later sutured. Special attention must be given no to include the ureteral mouths in the resection or suture. Most bladder surgeries can be accomplished by a regular expert gynecological laparoscopic surgeon.

When ureters are compromised, extraluminal nodules are usually easily removed by delicate surgery. If the ureteral wall is affected, a segmental resection is required, followed by immediate anastomosis, a complex surgery that should be performed only by expert laparoscopic surgeons. This is a rare location of the disease and is frequently accompanied by homolateral hydronephrosis of different degrees. Correct preoperative diagnosis is required. This can be accomplished by US (hydronephrosis), contrast MRI, and CAT (ureteral stops or deviations) scans. Double-“J” catheters after the anastomosis prevent filtrations and suture dehiscence.

Colorectal endometriosis is the most frequent location of DIE. Nodules can be found all along the rectosigmoid colon, from as near as the anal sphincter (rare) to as far as the lower sigmoid colon. Different techniques have been proposed over the years by many expert surgeons. Which one is appropriate in each case is the most important decision to be made, before or during laparoscopy.

In a recent review, Darai *et al*. ^87^ compared conservative surgery (disc resection) with radical surgery (segmental resection) in 31 patients. The conservative strategy had a shorter OR time and hospital stay ( *P* = 0.03 and *P* = 0.002, respectively) versus colorectal resection. Complication rates were similar for the two groups, but postoperative voiding was higher in the resection group.

Roman *et al*. ^88^ studied 122 consecutive patients managed by surgery in a follow-up that ranged from 1 to 6 years. The patients were treated with the authors’ technique of rectal shaving, in which the endometriotic nodule is dissected from the large bowel without compromising its structures. Sixty-eight patients were operated using US scalpel and 54 with plasma energy. In this group, two rectal fistulas occurred, and only 4.1% of the patients had recurrence of the disease. Roman *et al*. state that, with this evidence, rectal shaving is a valuable treatment with a low incidence of postoperative complications.

Ceccaroni *et al*. ^89^ recently presented a literature review that included 38 studies where they present the data regarding recurrence of colorectal endometriosis after surgery. A conflict they report is that the recurrence data vary among the studies, especially because there are different appraisals of what recurrence is and there are different lengths of follow-up reported (in general, 2 to 4 years). As suspected, in the longer studies, more recurrences occur.

Those authors cite recurrence rates that range from 2% to 43.5%. They found three risk factors for recurrence of colon DIE: age, body mass index (BMI), and type of surgery. Younger age is a risk factor. Higher BMI and incomplete surgery are the other two factors they identified.

In their review, they found four studies that correlated histopathological specimen margins (with disease or disease-free) with the risk of recurrence (38% with compromised margins).

In the lower rectal presentations (2–3 cm from the anal sphincter), transient colostomy is recommended. In the higher locations, this is rarely required. All procedures, in general, can be accomplished by laparoscopy. Interdisciplinary surgical teams are recommended except in those cases where gynecological laparoscopists routinely perform shavings, disc resections, or segmental colectomies (university hospitals and so on).

Zupi *et al*. ^90^ addressed the issue of pregnancy complications and DIE. In a multicenter, observational cohort study that included a group of women with incomplete previous surgery for endometriosis and a control group, they analyzed the results of spontaneous or ART pregnancies. With a media of 2 cm in diameter, the persistent nodules were linked to these obstetrical outcomes: higher risk of preterm delivery ( *P* = 0.0001), placenta previa ( *P* <0.0001), placental abruption ( *P* = 0.0392), and hypertension ( *P* = 0.0129).

**Figure 9.**
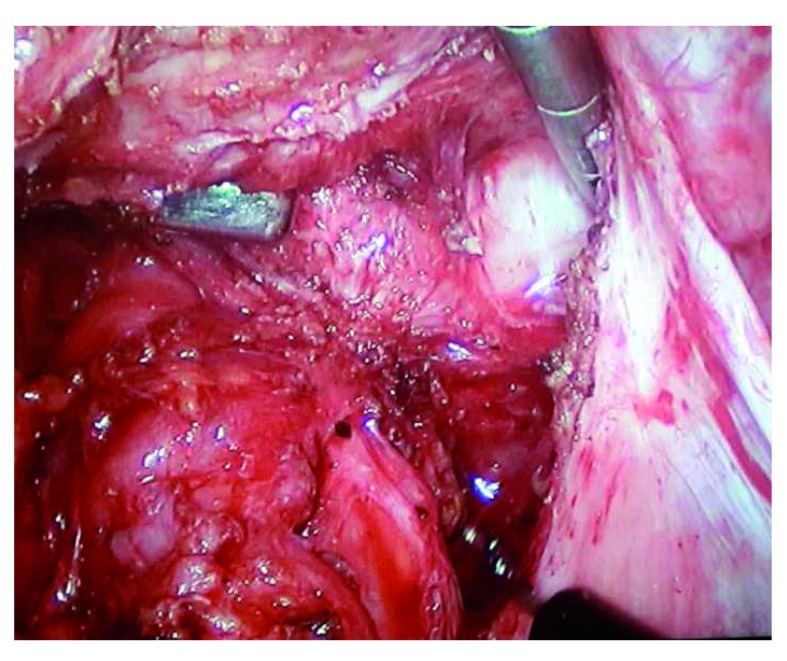
Colorectal image of deep infiltrating endometriosis after shaving of a nodule. Rectal shaving of endometriotic nodules has proven to be a safe and fast surgical procedure that alleviates pain and gastrointestinal symptoms even in low locations of the disease. Image is from surgery performed by the author.

Back in 2009, Brosens *et al*. ^91^ described a relationship between endometriosis and spontaneous hemoperitoneum in pregnancy (SHiP). They identified the disease as a major risk factor. In their literature review, which included 13 cases of endometriosis-associated SHiP, seven women had a diagnosis of DIE. The controversy on DIE and the need (or not) for surgery before seeking pregnancy should come to an end, and patients should be counselled on the risks of pregnancy prior to the resection of DIE nodules. Surgery is mandatory as a first step in the treatment of endometriosis-associated infertility in the presence of DIE ( Figure 9).

## How to understand endometriosis

After reading hundreds of publications on endometriosis, I realized that I began to understand this complex disease only when we started to share meetings and workshops with patients. No book, no online publication, no medical meeting can explain the intriguing pathways of endometriosis alone. Those who have it can tell a lot about it.

I have learned as much from them as from many extended articles written by enlightened authors. Science is not to be discarded. On the contrary, the facts and data it teaches give us the possibility to better understand those who have it.

The pain-ridden young woman who wanders from one doctor’s office to another, and then another, with no answers to her plight, should be the main subject of concern to those interested in endometriosis. To some extent, it is an entirely different medical specialty, not just a gynecological disorder.

As noted before, the disease probably starts at birth and could even be present at menopause. Pain usually starts at adolescence. At that age, girls skip school during menstrual periods in the most severe cases. Many of those who experience less pain are confronted with unexpected infertility later in life.

It is not just her. It is her family, her partner, and her daughters and sons who are confronted by the distress and disorientation linked to endometriosis. *Why me? How did I get it? How can I cope with it?*

**Figure 10.**
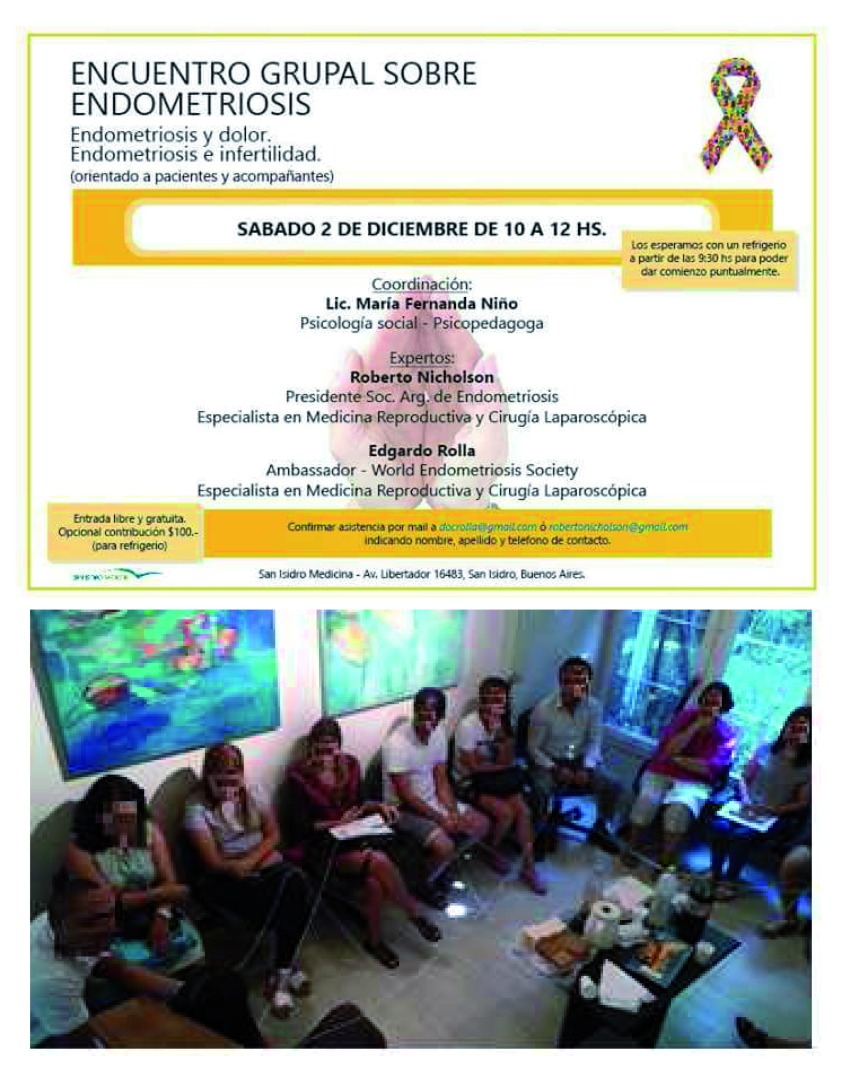
Poster inviting the public to attend a patients’ workshop with doctors and social psychologists and a photo from the workshop. Photo by the author.

Take your time, meet with patients’ groups and societies, accompany them in their public appearances, meet with them Saturday mornings at your office, and listen to them. In time, you will begin to understand endometriosis! As an example, let me share images from recent meetings and other activities with endometriosis patients held by our interdisciplinary professional team ( Figure 10– Figure 12).

**Figure 11.**
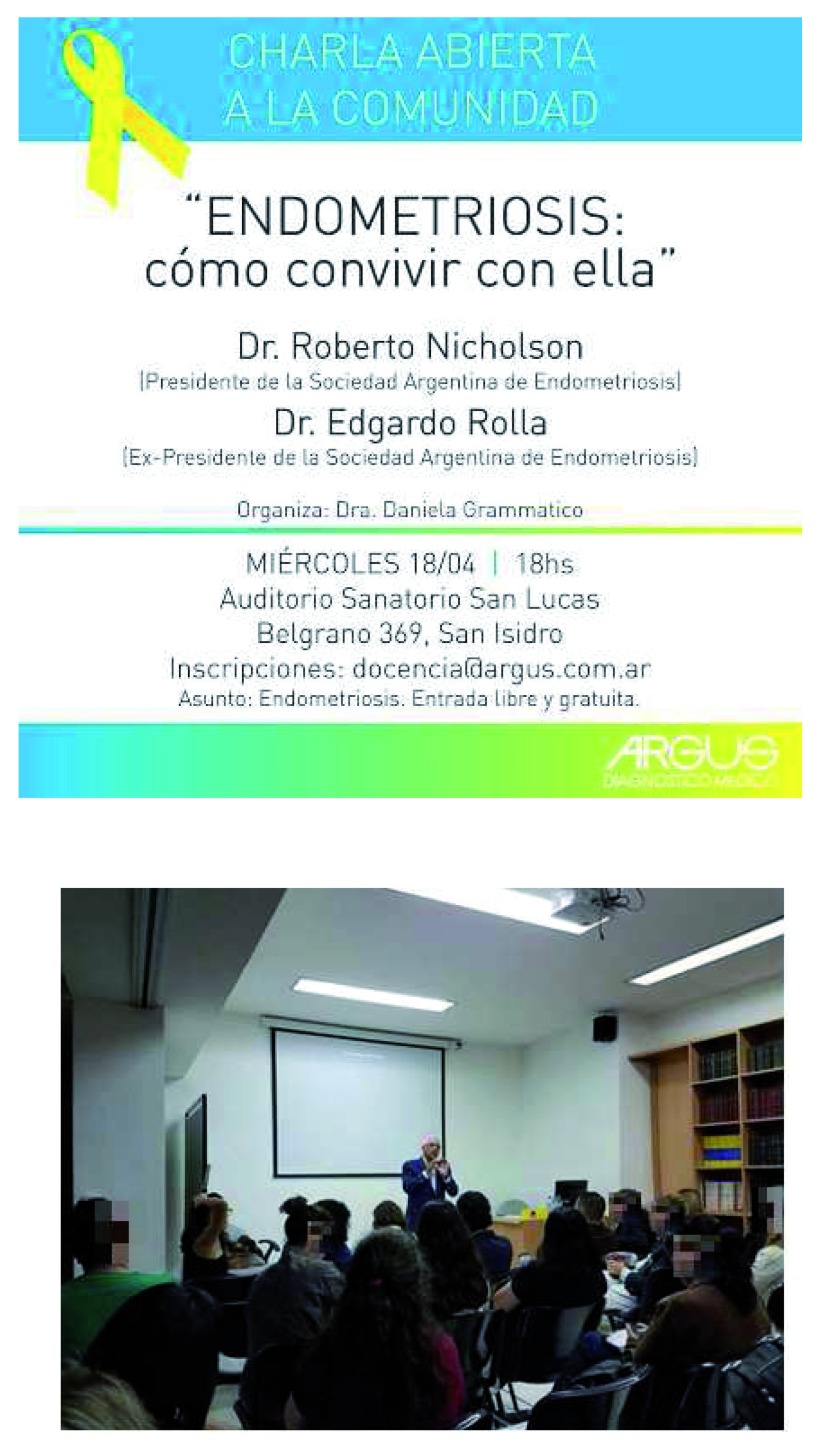
Poster inviting the public to attend a conference on endometriosis and a photo from the conference. Photo by the author.

**Figure 12.**
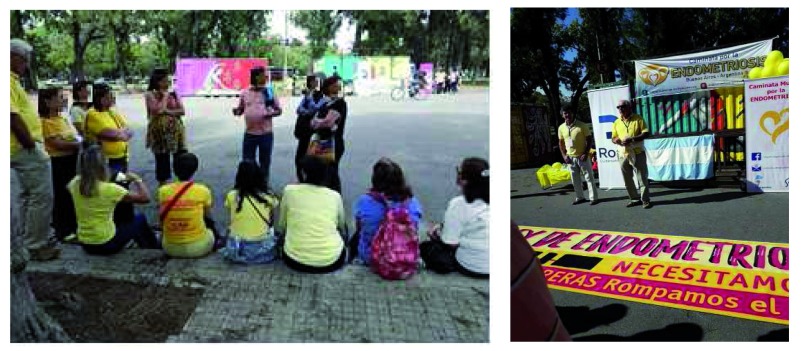
March for Endometriosis (2018). Public awareness talks at a park. Photos by the author.

## Conclusions

This is not a typical review article. It is not a systematic review of the literature or a meta-analysis. It is an updated literature-based publication. The bibliography was selected on a very personal basis. I have addressed pathogenesis, diagnosis, classification, and treatment by using mostly recently published articles as evidence. Some controversies arise from them, allowing readers to take sides, or not. The importance of understanding patients is crucial. Our medical actions should have patients as the center of our goals.
